# Use of the effluent from biogas production for cultivation of Spirulina

**DOI:** 10.1007/s00449-016-1726-2

**Published:** 2016-12-26

**Authors:** Malin Hultberg, Olle Lind, Göran Birgersson, Håkan Asp

**Affiliations:** 1 Department of Biosystems and Technology, Swedish University of Agricultural Sciences, P.O. Box 103, 230 53 Alnarp, Sweden; 2 Department of Plant Protection Biology, Swedish University of Agricultural Sciences, Alnarp, Sweden

**Keywords:** Anaerobic digestate effluent, *Arthrospira platensis*, Biofertiliser, Microalgae, Nutrient recycling

## Abstract

The effluent from the biogas process was tested as a nutrient source during cultivation of the protein-rich and edible microalgae Spirulina (*Arthrospira platensis*) and compared with conventional Spirulina medium. Equal biomass production was observed until late exponential phase and no significant differences could be observed between the treatments in protein amount, amino acid composition, and total lipid concentration. The concentration of the pigment phycocyanin differed significantly between Spirulina medium and the effluent-based medium (63.3 ± 11.7 and 86.2 ± 1.9 mg g^−1^, respectively). Slightly higher concentrations of saturated fatty acids, mainly palmitic acid, were observed in the biomass produced in Spirulina medium than in that produced in the effluent-based medium. In the biomass produced in the effluent-based medium, the cadmium concentration was 0.07 ± 0.05 mg kg^−1^ of dry weight, whereas it was below the detection limit in the biomass produced in Spirulina medium. There is a need to identify new food and feed resources and a possible future scenario is to integrate Spirulina production into the biogas plant for protein production as it contains more than 60% of protein on dry weight basis. In that scenario, it is important to control heavy metal concentrations in the biogas slurry fed to Spirulina.

## Introduction

The world’s population is increasing rapidly, and there is a need to identify new food and feed resources. One possibility to meet the need for high-quality protein is through increased production of the microorganism known as Spirulina. Taxonomically, Spirulina generally comprises the species *Arthrospira platensis* and *A. maxima* within the genus *Arthrospira* [1]. However, the term Spirulina is commonly used for historical reasons. Spirulina is a multicellular edible cyanobacterium, capable of photosynthesis, that is traditionally used as a food source in Mexico and parts of Africa. Dried Spirulina biomass contains approximately 60% protein, which is high compared with traditional protein crops, such as soybean. Spirulina contains all essential amino acids and also high-quality lipids, with a substantial amount of polyunsaturated fatty acids [2]. It should also be pointed out that in contrast to traditional crops, such as soybean, Spirulina does not require arable land for its production. Commercial production is normally performed in open ponds [3]. Spirulina is, therefore, suitable for food production in the vicinity of nutrient-rich wastewater streams, regardless of the availability of soil, e.g., in urban or peri-urban areas.

During production of Spirulina, and also of other photosynthetic microorganisms, macronutrients, such as nitrogen and phosphorus, are commonly supplied in the form of commercial inorganic fertiliser. Replacing these inorganic chemical compounds with residue streams would increase both sustainability and economics during production [4]. An important aspect is that global phosphorus reserves are declining, and thus, phosphorus recycling is crucial [5]. Therefore, the economic feasibility of Spirulina production on organic waste sources can be expected to increase when the supply of inorganic phosphorus decreases. Studies on cultivation of Spirulina with residue streams as the nutrient source have generally reported good results [6]. A potential explanation for this is that cultivation of Spirulina has traditionally been based on photoautotrophy. However, higher growth rate has been demonstrated under conditions allowing mixotrophic growth [7]. This type of growth, where the microorganisms retrieve energy from organic carbon sources and also from light, can be expected in a medium based on residue streams, since mineral nutrients and organic carbon will be present.

In parallel with the increase in the world’s population, there is a growing need for energy and increased interest in renewable energy sources [8]. One alternative is anaerobic waste treatment, a net energy producing technology through production of biogas during anaerobic digestion of the waste. This technology is now used on a large scale in many countries for treatment of different types of agro-industrial wastes [9]. Depending on the initial substrate/s added in the anaerobic process, the effluent from the biogas process is often transferred back to agriculture and used as a biofertiliser. At small-scale level, biogas plants have been integrated with greenhouse crop production through the multifunctional use of anaerobic digestion reactors, in which heating from combustion of the methane produced and fertilisation with digestate and carbon dioxide have synergistically increased the productivity of the system [10, 11]. The same principle of integration could possibly be applicable to production of Spirulina, through the use of digestate as a nutrient source and methane combustion for heating and carbon dioxide biofixation in closed photobioreactors [12]. Furthermore, tubular photobioreactors would allow for high light and space use efficiency due to the large illumination surface and vertical orientation [13], making such a Spirulina biomass producing system suitable in an urban setting, where production of biodegradable waste is high.

In the present study, the effluent from a biogas plant, processing plant residues only, was tested as a nutrient source during cultivation of Spirulina and compared with a conventional Spirulina medium. The growth rate of Spirulina and biochemical factors in the biomass, such as protein concentration and amino acid composition, lipid composition, and production of phycocyanin, were analysed. Accumulation of cadmium was also determined, as the presence of potential contaminates and their risk for transmission into the food chain must be considered in any residue-based process.

## Materials and methods

### Microorganism and media

*Spirulina platensis* LB 2340 was obtained from UTEX Culture Collection of Algae at the University of Texas, Austin, USA. The strain was routinely cultivated in Spirulina medium with the following composition: (g L^−1^) NaHCO_3_ 13.6, Na_2_CO_3_ 4.0, K_2_HPO_4_ 0.5, NaNO_3_ 2.5, K_2_SO_4_ 1.0, NaCl 1.0, MgSO_4_·7H_2_O 0.2 g, CaCl_2_·2H_2_O 0.04 g, (mg/l) Na_2_EDTA·2H_2_O 4.60, FeCl_3_·6H_2_O 0.60, MnCl_2_·4H_2_O 0.26, ZnCl_2_ 0.03, CoCl_2_·6H_2_O 0.03, Na_2_MoO_4_·2H_2_O 0.04, CuSO_4_·5H_2_O 0.02, ZnSO_4_·7H_2_O 0.04, H_3_BO_3_ 0.06, and cyanocobalamin 0.14. The composition corresponded to the following concentration of macronutrients: (mg L^−1^) N–NO_3_ 411.6, P 89.0, K 673.2, Ca 10.9, S 210.2, and Mg 19.7.

Effluent from the biogas process was obtained from Jordberga Biogas, Trelleborg, a commercial biogas plant in southern Sweden producing biogas and biofertiliser based on plant residues obtained from local agriculture. The effluent was filtered in polyamide filters (Sintab AB, Sweden) in several steps with a final mesh size of 5 µm to remove particles. The concentrations of nitrate and ammonium in the filtered effluent were determined colorimetrically on an automatic analyser (TRAACS 800 Bran-Luebbe) using the Berthelot reaction for ammonium and the cadmium reduction method for nitrate [14]. The concentrations of phosphorus and other nutrients were analysed by inductively coupled plasma optical emission spectrometry (ICP-OES) according to ISO 11885.

The filtered effluent was used in a carbonate buffer, pH 9.2, composed of 13.6 g L^−1^ NaHCO_3_ and 4.0 g L^−1^ Na_2_CO_3_. In the experiments, a concentration of 1.5% (v/v) of the effluent was added at the start as a nutrient source, a similar volume of effluent was added after 3 days of growth, and a final addition of 3% (v/v) of the initial volume was added after 6 days of growth. Thus, a total of 6% effluent was used in the medium, which corresponded to an added macronutrient concentration of: (mg L^−1^) N–NH_4_ 93.0, P 3.9, K 93.0, Ca 16.9, S 3.5, and Mg 2.1. The effluent was added in a stepwise manner to allow light transmission during algal growth.

### Experimental setup

The experimental setup included the effluent-based medium described above and the control treatment based on Spirulina medium. All experiments were performed in a greenhouse with a 16 h/8 h day/night regime with an added light intensity of 100 µmol m^−2^ s^−1^ and the temperature set to 25 °C. The experiments were performed as batch cultures with a total volume of 3 L and were continuously stirred at a speed of 100 rpm. For inoculation, an inoculum size comprising 10% (v/v) of a 7-day-old culture cultivated in Spirulina medium was added. The inoculum corresponded to 230 ± 6 mg L^−1^ dry weight (DW) of biomass. For evaluation of biomass productivity, samples were taken on days 3, 5, 7, and 10. For evaluation of protein concentration, amino acid composition, total lipids and lipid quality, production of phycocyanin and allophycocyanin and cadmium uptake, and samples were taken on day 6. These analyses are described in the following.

## Analyses

### Biomass production

The algal biomass was collected by filtration through nylon filters with a mesh size of 10 µm and washed once with an equal amount of distilled water. Before and after filtration, the filters were dried in an oven at 60 °C until constant weight, to determine the dry weight of the algal biomass collected.

### Biochemical composition

For analysis of the biochemical composition, the algal biomass was harvested at day 6 by filtration as described above. The washed biomass was then frozen at −80 °C, freeze-dried under vacuum, and used for the analysis described in the following.

### Protein concentration and amino acid composition

Total amount of protein was analysed by the Dumas method [15] using a Thermo Scientific™ FLASH 2000 CHNS/O Analyzer. Amino acid composition, including alanine, arginine, aspartic acid, cysteine, glutamic acid, glycine, histidine, isoleucine, leucine, lysine, methionine, phenylalanine, proline, serine, threonine, tyrosine, and valine, was determined at a certified laboratory (Eurofins Food & Agro Testing Sweden AB, Linköping, Sweden) by ion-exchange chromatography according to the method by Llames and Fontaine [16].

### Fatty acid methyl ester (FAME) content analysis

The lyophilised algal biomass was treated with methanolic H_2_SO_4_ (2% v/v) for 60 min at 90 °C. Fatty acid methyl esters (FAME) were then extracted with hexane and analysed by combined gas chromatography and mass spectrometry (GC–MS; Agilent 6890 GC and 5975 MS—Agilent Technologies, Santa Clara, CA, USA). The GC was equipped with a 60 m fused silica capillary column (ID 0.25 mm) coated with HP-5MS UI (Agilent Technologies). Aliquots comprising 2 µL of sample were injected by an auto-injector (Agilent 7683B; Agilent Technologies) at 250 °C. The GC oven was programmed at 125 °C for 2 min, followed by an increase of 4 °C min^−1^ up to 250 °C and isothermal for 10 min, and a post-run cleaning of the column at 275 °C for 1 min. The mass spectra were generated at 70 eV, acquiring data over m/z 29–400 at a scanning rate of 1.99 scans s^−1^. For quantification, heptadecanoic acid was added before esterification as an internal standard. Samples without the addition of heptadecanoic acid were also prepared, since the presence of this fatty acid has been reported in some strains of *Arthrospira* [17]. The amounts of FAME in the samples were based on total ion chromatogram (TIC) peak area with exception of the non-separating C18:1, C18:2, and C18:3, and the amounts of which were determined by quantification of molecular ions, using extracted ion chromatograms.

### Determination of phycocyanin

The lyophilised samples were mixed with phosphate buffer, pH 7, using the method described by Chainapong et al. [18]. The equations based on spectrophotometric readings at 618 and 650 nm described by Kursar and Alberte [19] were applied to the results.

### Cadmium content

Lyophilised Spirulina biomass produced in Spirulina medium, in effluent-based medium and also the freeze-dried filtered effluent was wet-combusted in HNO3 (65%) using a microwave technique. The concentration of cadmium in the samples was analysed by the commercial laboratory LMI AB, Sweden using analytical method SS 028150-2 based on ICP-MS, detection limit 0.1 µg L^−1^.

### Statistical analysis

Each experiment was carried out in triplicate and repeated once, and mean values and standard deviation (SD) are reported. The data were analysed by analysis of variance followed by Tukey’s multiple comparison test. Differences were considered significant at *P* < 0.05 (Minitab, version 16).

## Results

### Biomass production

**Fig. 1:**
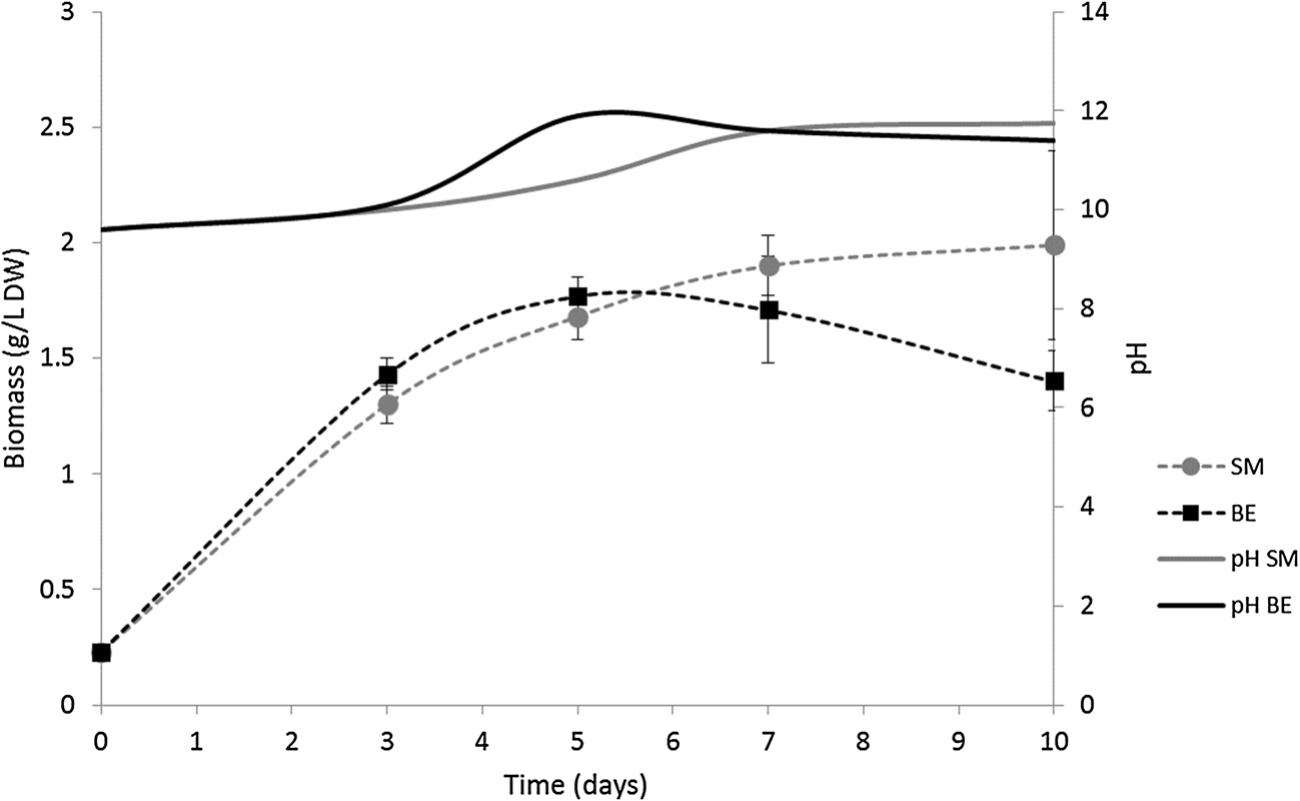
Growth (g L^−1^ dry weight, DW) of Spirulina (*Arthrospira platensis*) in effluent-based medium (BE) and in Spirulina medium (SM), and pH changes in the medium over time

As shown in Fig. 1, the biomass production observed in the effluent-based medium was equal to that in Spirulina medium during the first 6 days. Thereafter, a decrease in biomass was observed in the effluent-based medium, whereas the amount of biomass in Spirulina medium remained stable. The pH increased to a higher value between day 3 and day 7 during growth in the effluent-based medium.

### Biochemical composition

#### Protein concentration and amino acid profile

Total concentration of protein, expressed as % of DW, in the biomass harvested after 6 days of growth was 60.5 ± 6.2 and 63.3 ± 2.7 for Spirulina medium and effluent-based medium, respectively, and no significant differences were observed between the treatments. Table 1 shows the concentrations of individual amino acids in the biomass, with high concentrations of glutamic acid, aspartic, and leucine. No significant differences were observed in amino acid composition between the treatments.

**Table 1:** Amino acid composition (g 100 g^−1^ protein, DW basis) of Spirulina (*Arthrospira platensis*) grown on Spirulina medium (SM) and on a medium based on filtered effluent from a biogas plant (BE)

Amino acid	SM	BE
Alanine	4.67 ± 0.60	4.53 ± 0.37
Arginine	3.96 ± 0.54	3.91 ± 0.31
Aspartic acid	6.05 ± 0.68	5.81 ± 0.40
Cysteine	0.43 ± 0.05	0.43 ± 0.04
Glutamic acid	8.08 ± 0.91	7.65 ± 0.50
Glycine	3.06 ± 0.35	2.99 ± 0.24
Histidine	0.99 ± 0.14	0.97 ± 0.09
Isoleucine	3.32 ± 0.46	3.23 ± 0.28
Leucine	5.35 ± 0.66	5.19 ± 0.43
Lysine	2.96 ± 0.38	2.91 ± 0.26
Methionine	1.41 ± 0.19	1.47 ± 0.16
Phenylalanine	2.83 ± 0.33	2.70 ± 0.20
Proline	2.24 ± 0.20	2.20 ± 0.12
Serine	3.24 ± 0.30	3.06 ± 0.22
Threonine	3.34 ± 0.29	3.23 ± 0.28
Tyrosine	2.55 ± 0.35	2.56 ± 0.16
Valine	3.75 ± 0.52	3.62 ± 0.30

No significant difference was found between the treatments

#### Fatty acid methyl ester (FAME) content analysis

Total concentration of lipids in the biomass harvested after 6 days of growth was 34.8 ± 3.8 and 42.0 ± 4.2 µg mg^−1^ DW for Spirulina medium and effluent-based medium, respectively, and no significant differences were observed between the treatments. Significant differences were observed in the fatty acid profile of the different treatments, as shown in Table 2. A slightly higher concentration of saturated fatty acids, mainly palmitic acid, was observed in the biomass produced in Spirulina medium than in the biomass produced in the effluent-based medium. The biomass produced in the effluent-based medium had slightly higher concentrations of the unsaturated fatty acids: palmitoleic acid and oleic acid. Heptadecanoic acid was not naturally present in the biomass, but 2-hydroxyheptadecanoic acid was detected in both treatments. Low concentrations of the long-chain fatty acids: arachidic acid and eicosatrienoic acid were also detected in the biomass.

**Table 2:** Relative proportions of fatty acids (% of total fatty acid amount) in biomass of Spirulina (*Arthrospira platensis*) when grown on Spirulina medium (SM) or on a medium based on filtered effluent from a biogas plant (BE)

Fatty acid	SM	BE
14:0	0.3a*	0.4a
15:0	0.4a	0.3a
16:0	42.8a	38.6b
16:0 2OH	0.5a	0.2a
16:1	2.8a	4.5b
17:0 2OH	2.3a	3.3b
17:1	0.1a	0.4b
18:0	5.3a	3.4a
18:1	8.8a	10.2b
18:2	26.9a	28.7a
18:3	9.8a	9.7a
20:0	0.06a	0.1a
20:3	0.04a	0.2b
ΣSFA	51.6	46.3
ΣUFA	48.4	53.7

* Values within rows followed by different letters are significantly different (*P* < 0.05, Tukey’s test)

#### Determination of phycocyanin and allophycocyanin

The concentration of the pigment phycocyanin differed significantly between Spirulina medium and the effluent-based medium (63.3 ± 11.7 and 86.2 ± 1.9 mg g^−1^, respectively). For allophycocyanin, no significant difference in concentration was observed (37.1 ± 7.4 and 41.3 ± 2.1 mg g^−1^, respectively).

#### Cadmium content

Cadmium concentration was below the detection limit in the biomass produced in Spirulina medium. However, in the biomass produced in the effluent-based medium, the cadmium concentration was 0.07 ± 0.05 mg kg^−1^ DW. The filtered effluent used in the study had a cadmium concentration of 0.36 ± 0.01 mg kg^−1^ DW.

## Discussion

This study shows that the anaerobic digestate effluent produced during the biogas process can be used for production of Spirulina. Compared with the Spirulina medium, which is optimized considering both nutrient composition and buffer capacity, equal biomass production was observed until late exponential phase. Furthermore, there were no significant differences in protein amount and amino acid composition of the biomass produced between the treatments. The total amount of protein and the amino acid composition observed agree well with previous findings [2, 20] and confirm the potential of Spirulina as a source of high-quality protein. The total amount of lipids was slightly, although not significantly, higher in the biomass produced in the effluent-based medium. Some differences in lipid composition were observed between the different treatments, with a slightly higher concentration of unsaturated fatty acids in the biomass produced in the effluent-based medium. Overall, the lipid composition agreed well with previous findings on this strain [17], with a dominance of palmitic acid followed by linoleic acid.

The nutrient concentration and quality differed widely between the treatments and the decline in biomass observed in the stationary phase in the effluent-based medium could possibly have been avoided if a higher nutrient level had been applied. However, adding the filtered effluent in the amount needed to reach the nutrient concentrations present in Spirulina medium is not an option for cultivation of phototrophic organisms, since the effluent from the biogas process is characterised by high turbidity and high ammonia content [21]. Both these parameters have a negative effect on photosynthesis, turbidity by decreasing penetration of light, and ammonia by an uncoupling effect on the photosynthesis process [22]. A significant increase in phycocyanin concentration in the effluent-based medium was observed in the present study and might be related to the higher turbidity compared with Spirulina medium. Phycocyanin has a role as an accessory pigment and increased production could have been a physiological response to the lower amount of light. Similarly, Jung et al. [23] who used extracts from oyster shells and soil as an amendment to Zarrouk’s medium during cultivation of Spirulina (*A. maxima*) observed a significant increase in phycocyanin concentration when the extract was added.

The ammonium concentration in anaerobic effluent has also been demonstrated to be a limiting factor in trials on use of digestate in hydroponic plant cultivation [24, 25]. To enable a higher nutrient concentration to be provided, ammonia stripping and nitrification has been used as a means to avoid ammonia toxicity at low dilution levels of digestate [26]. Furthermore, nitrification of anaerobic effluent has been shown to lead to rapid sedimentation of suspended solids, providing a clear, yellowish liquid [27]. This would be desirable to avoid turbidity during Spirulina cultivation. Moreover, the high pH applied for cultivation of Spirulina can be expected to cause considerable ammonia volatilisation, similar to the ammonia emissions issue reported for soil fertilisation with digestate [28]. Nitrification pre-treatment would mitigate these emissions through decreasing the ammonia:nitrate ratio, but might lead to substantial emissions of nitrous oxide during bacterial oxidation of ammonia [29].

The cyanobacteria generally have high environmental tolerance and are able to use a wide range of nitrogen sources, including ammonium, nitrate, and organic compounds, such as amino acids, with ammonium as the preferred nitrogen source [30]. In the present study, in Spirulina medium, nitrogen was supplied as nitrate in an initial concentration of 412 mg L^−1^, whereas in the effluent-based medium, nitrogen was mainly supplied as ammonium in a total concentration of 93 mg L^−1^. The amount of protein in Spirulina biomass has been reported to be affected by the nitrogen concentration [31]. However, this was not observed in the present study, where there was no difference in either crude protein content or amino acid composition between the treatments. As pointed out by Markou and Georgakakis [4], contradictory results have been reported concerning the effect of nitrogen concentration on the quantity and quality of lipids in Spirulina biomass. In the present study, a slight increase in total amount of lipids was observed in the biomass produced in the effluent-based medium. However, phosphorus was supplied in a considerably lower amount in the effluent-based medium (4 mg L^−1^) than in the optimized medium (89 mg L^−1^), which could have affected this result, because P (and N) deficiency has been reported to increase lipid production in macroalgae [32].

The presence of potential contaminants and their risk of transmission into the food chain is an important parameter to consider in all residue-based processes. In the present study, the cadmium concentration was below the detection limit in the biomass produced in Spirulina medium. However, in the biomass produced in the effluent-based medium, the cadmium concentration was 0.07 ± 0.05 mg kg^−1^ DW. The filtered effluent used in the study had a cadmium concentration of 0.36 ± 0.01 mg kg^−1^ DW. Pollution of the environment by heavy metals due to industrial and agricultural activities is well documented and has occurred worldwide over the past two centuries [33]. It should also be pointed out that there are soils, e.g., alum shale, with a naturally high concentration of heavy metals, such as cadmium, which adds to the final soil cadmium concentration [34]. The concentration of heavy metals in the effluent from biogas production will vary depending on the substrates used in the process [9]. In Sweden, the concentration of cadmium in effluent from the biogas process is monitored and a range of 0.3–0.6 mg kg^−1^ dry matter is commonly reported [35]. The concentration in the biogas effluent used in the present study (0.36 ± 0.01 mg kg^−1^ DW) was within this range. Microalgae and also the cyanobacterium Spirulina are known to have a high capacity for accumulation of heavy metals in their biomass [36]. Accumulation of cadmium was confirmed in the present study, with a mean concentration of 0.07 ± 0.05 mg kg^−1^ Spirulina dry matter produced in the effluent-based medium, whereas the concentration was below the detection limit for the biomass produced in Spirulina medium. However, the amount of cadmium observed in the biomass produced in the effluent-based medium was still considerably below the established threshold for food [37].

From the present study, it can be concluded that the effluent from the biogas process sustained good growth of Spirulina and, what is more important, that the quality of the biomass was high. As pointed out by de Groot and Bogdanski [9], there is increasing interest in anaerobic digestion of farm and household residues, e.g., smallholder biogas digesters and community biogas plants can be found worldwide. An interesting future scenario is, therefore, to integrate Spirulina production into the biogas plant. Simplified Spirulina production techniques, similar to simplified hydroponic techniques promoted for increased food security and economic stability in poor urban areas [38], could be developed and integrated with small-scale low-cost biogas plants. This scenario implies a need for identifying and using other residue streams from the process, such as residual heat. As demonstrated in the present study, the heavy metal concentration in the biogas slurry is an important parameter to control. It should also be pointed out that the components used in the effluent-based medium in the present study were not subjected to any kind of disinfection or sterilisation treatment, which would facilitate establishment of a large-scale process.
